# Exploration of Functional Connectivity During Preferred Music Stimulation in Patients with Disorders of Consciousness

**DOI:** 10.3389/fpsyg.2015.01704

**Published:** 2015-11-09

**Authors:** Lizette Heine, Maïté Castro, Charlotte Martial, Barbara Tillmann, Steven Laureys, Fabien Perrin

**Affiliations:** ^1^Coma Science Group, GIGA & Cyclotron Research Center and Neurology Department, University and University Hospital of LiègeLiège, Belgium; ^2^Auditory Cognition and Psychoacoustics Team – Lyon Neuroscience Research Center (UCBL, CNRS, UMR 5292, INSERM U1028)Lyon, France

**Keywords:** music, disorders of consciousness, fMRI, functional connectivity, auditory network, external network

## Abstract

Preferred music is a highly emotional and salient stimulus, which has previously been shown to increase the probability of auditory cognitive event-related responses in patients with disorders of consciousness (DOC). To further investigate whether and how music modifies the functional connectivity of the brain in DOC, five patients were assessed with both a classical functional connectivity scan (control condition), and a scan while they were exposed to their preferred music (music condition). Seed-based functional connectivity (left or right primary auditory cortex), and mean network connectivity of three networks linked to conscious sound perception were assessed. The auditory network showed stronger functional connectivity with the left precentral gyrus and the left dorsolateral prefrontal cortex during music as compared to the control condition. Furthermore, functional connectivity of the external network was enhanced during the music condition in the temporo-parietal junction. Although caution should be taken due to small sample size, these results suggest that preferred music exposure might have effects on patients auditory network (implied in rhythm and music perception) and on cerebral regions linked to autobiographical memory.

## Introduction

Patients with disorders of consciousness (DOC) are a patient population that is very difficult to assess. Following coma, these patients can be in an unresponsive wakefulness syndrome (UWS) where behavior is reflexive, and awareness of the self and surrounding is absent (The Multi-Society Task Force of Pvs, 1994; Laureys et al., 2010), or in a minimally conscious state (MCS) where behaviors indicating awareness are limited, fluctuating but reproducible (Giacino et al., 2002). Various interferences, both physical and cognitive impairments, or medical complications can affect the diagnosis based on clinical assessments of consciousness (Schnakers et al., 2009). This is one of the issues underlying the current misdiagnosis rate of 40% (Schnakers et al., 2009; van Erp et al., 2015). Consequently, numerous research is investigating the neural and cerebral responses of these patients, with the aim to provide unbiased and objective measures complementing bedside evaluation and helping diagnosis (Laureys and Schiff, 2011; Stender et al., 2014).

Previous research has also proposed to increase the sensitivity of clinical tests by using personally relevant stimuli (Perrin et al., 2015). For example, several behavioral studies have shown that a higher number of responses could be observed following self-referential stimuli, like the use of a mirror or the patient’s own name, as compared to neutral stimuli (Vanhaudenhuyse et al., 2008; Cheng et al., 2013; Di et al., 2014). Neurophysiological studies have indicated that salient and emotional stimuli increase the probability of observing a cerebral response in patients with DOC. For example, the probability to observe a P300 event-related response (i.e., a brain response reflecting stimulus processing) is enhanced when the deviant stimulus is not a tone stimulus but the patient’s own name (Perrin et al., 2006; Cavinato et al., 2011). Very recently, it has also been shown that preferred music (i.e., an autobiographical and emotional stimulus) has an effect on cognitive processes of patients with DOC. Indeed, observing a P300 to one’s own name was increased in patients with DOC after having been exposed to their preferred music compared to a control condition (i.e., acoustically similar noise; Castro et al., 2015). This result is in agreement with a study showing increased behavioral responses after preferred music (Verger et al., 2014), and several single-case studies with DOC patients suggesting effects of music on a behavioral level (Magee, 2005; Magee et al., 2014).

Resting state functional MRI allows investigation of several distinct, reproducible and dynamic brain networks (Beckmann et al., 2005; Damoiseaux et al., 2006; De Luca et al., 2006; Laird et al., 2011), without the need for patients’ cooperation (Soddu et al., 2011). The auditory network is one of the reliably observed networks, even though not yet extensively studied. This network encompasses primary auditory cortices including Heschls gyri, superior temporal gyri, insula, cingulate, post- and pre-central gyri, and supramarginal gyrus (Beckmann et al., 2005; Smith et al., 2009; Laird et al., 2011). The auditory network can be observed in 81% of healthy subjects, 46% in MCS, and is limited to 21% of UWS patients (Demertzi et al., 2014). In fact, it has strong power to discriminate MCS and UWS patients, making automatic classification possible (Demertzi et al., 2015). Another network that is also related to auditory processing (Brunetti et al., 2008) is the external network. This network is also related to external orientation, goal-directed behaviors, and cognitive processing of somatosensory (Boly et al., 2007), and visual (Dehaene and Changeux, 2005) input. The external network is often named the ‘dorsal attention network,’ or ‘task positive’ network (Greicius et al., 2003; Vanhaudenhuyse et al., 2010a). It has been shown to be anticorrelated with an internal/default mode network (Greicius et al., 2003; Vanhaudenhuyse et al., 2010a), implicated in self-awareness and stimulus-independent thoughts in healthy controls (Raichle et al., 2001; Greicius et al., 2009). Interestingly, auditory, external and internal/default mode networks include cortical regions that have been shown to be modulated by emotional sounds. Indeed, as compared to noise, meaningful sounds (infant cries or the patient’s own name) are associated to a widespread activation of the auditory cortex and medial cortical structures in DOC patients (Laureys et al., 2004). Thus, the effect of music as reported in Castro et al. (2015) is probably also associated to functional connectivity changes of these regions.

We here aim to explore whether the effect of music in severely brain-damaged patients with DOC is related to functional connectivity changes. Functional MRI scans were acquired while participants were exposed to their preferred music as well as a control condition when they were exposed to the repetitive noise from the scanner (also present in the music condition). Using a functional connectivity parcellation (Gordon et al., 2014), we assessed functional connectivity using seed regions in both primary auditory cortices. We also analyzed network connectivity of the auditory network, the external network, and default mode network. We expect to observe changes, and more specifically increases, in functional connectivity in the auditory and attentional systems in patients with DOC during the music stimulation (vs. the control condition).

## Materials and Methods

### Participants

Eight healthy participants (four female; mean age = 26 years, *SD* = 3), and seven patients (four MCS; three UWS) were scanned between March 2014 and April 2015 for this study. Patients were excluded for this study when any contraindication for MRI was present (e.g., presence of ferromagnetic aneurysm clips, pacemakers), or when patients needed sedation. Chronic patients with DOC were hospitalized for 1 week of assessment at the coma science group, University hospital of Liege, Belgium. Multiple behavioral assessments in the form of the CRS-R were completed, including one the morning before the (f)MRI acquisition. One patient showed drain artifacts on the T1 and functional MRI scan covering more then 40% of the brain, and in one patient the segmentation could not be reliably performed due to the lesion extent. Our patient population consisted thus of five patients (three MCS, two UWS; mean age = 50 years, *SD* = 10; **Table 1**). The ethics committee of the medical school of the University of Liège approved the study.

**Table 1:** Diagnostics of the five patients with disorders of consciousness (DOC).

		DOC1	DOC2	DOC3	DOC4	DOC5
Sex		Male	Female	Female	Male	Male
Age (years)		40	50	39	61	58
Time since injury (months)		12	6	26	13	25
Etiology		Trauma	Anoxic	Trauma	Anoxic	Anoxic
Diagnosis		UWS	UWS	MCS -	EMCS	MCS +
CRS-R score	A.	1	1	2	4	3


	V.	0	0	3	5	0
	M.	2	1	2	6	1
	O.	0	1	1	3	1
	C.	0	0	0	2	0
	Ar.	1	2	2	3	2
	Total	4	5	10	23	7
Structural MRI		Subcortical diffuse axonal injury, moderate enlargement of the ventricles, and atrophy of midbrain and sulci	Cortical and subcortical atrophy with severe post-anoxic leukoencephalopathy	Right lenticular lesion, diffuse axonal injury, and enlargement of the third ventricles	Extensive defects in region of the posterior cerebral artery, thalamus, and enlargement of right lateral ventricle	Global hemosiderosis and ischemic damage, white matter intensities (frontal + temporal), and enlargement of the ventricles
Neuroimaging (PET)		Indicated MCS	Consistent with an UWS	Consistent with MCS	Consistent with EMCS	Consistent with MCS

*CRS-R, coma recovery scale revised; A., auditory function; V., visual function; M., motor function; O., oromotor/verbal function; C., communication; Ar., arousal; UWS, unresponsive wakefulness syndrome; MCS, minimally conscious state; EMCS, emergence from minimally conscious state.*

### Music Stimulation and Procedure

Five musical excerpts were selected for each participant from a questionnaire on musical preference completed by family members or loved ones (for the patients) or the participant him/her self (for the healthy participants). These musical excerpts had a mean duration of 2 min and were all dynamic, musically coherent, and representative of the whole musical piece. The five excerpts were combined to create a musical stimulus of a duration of 10 min and 10 s, which overlaps with the duration of the functional scan. Fading in and fading out (around 2 s) was added to avoid rough transitions between the excerpts.

The functional scan was acquired twice during one MRI scanning session. Once with the participants’ preferred music (i.e., music condition), and once when participants were exposed to the repetitive noise from the scanner (i.e., control condition). This control condition is the same as used for the investigation of a classical resting state. The order of the conditions was randomized between participants, and the two functional scans were always separated by a delay of 10 min to reduce any potential order effects. Instructions and musical stimuli were delivered through MR compatible Siemens headphones. Participants were instructed to keep their eyes closed, stay awake, avoid any structured thoughts, and listen attentively to the music.

### MRI Acquisition and Analysis

Two sets of 300 T2^∗^-weighted images were acquired using a 3T Siemens TIM Trio MRI scanner (Siemens Medical Solutions, Erlangen, Germany) with a gradient-echo echo-planar imaging sequence using axial slice orientation and covering the whole brain (32 slices; voxel size = 3 mm × 3 mm × 3 mm; matrix size = 64 × 64 × 32; repetition time = 2000 ms; echo time = 30 ms; flip angle = 78°; field of view = 192 mm × 192 mm). The 10 initial volumes were discarded to avoid T1 saturation effects. Data preprocessing was performed using Statistical Parametric Mapping 8 (SPM8^1^). Preprocessing steps included realignment and adjustment for movement-related effects, slice time correction, co-registration of functional onto structural data, segmentation of structural data, spatial normalization of all data to standard stereotactic Montreal Neurological Institute (MNI) space using the normalization parameters which had resulted from the segmentation step. Normalized functional data were then smoothed using a Gaussian kernel with an isotropic 8 mm of full-width half-maximum.

Motion correction was applied using an automatic artifact detection tool for global mean and motion outliers^2^. Outliers in the global mean signal intensity and motion were identified and included in the subsequent statistical analysis as nuisance parameters (i.e., one regressor per outlier within the first-level general linear models). Specifically, an image was defined as an outlier (artifact) image if the head displacement in x, y, or z direction was greater than 0.5 mm from the previous frame, or if the rotational displacement was greater than 0.02 radians from the previous frame, or if the global mean intensity in the image was greater than 3 SD from the mean image intensity for the entire resting session. For our group of patients, the number of motion outlier images did not differ significantly between music and noise sessions (two-sided paired *t*-test; *p* = 0.16, music condition *m* = 16, *SD* = 18; control condition *m* = 3, *SD* = 4). Healthy participants did not show any movement-affected outlier scans.

Analyses of functional connectivity were performed using the connectivity toolbox “conn,” version 15D^3^ (Whitfield-Gabrieli and Nieto-Castanon, 2012). As recently recommended (Behzadi et al., 2007; Murphy et al., 2009; Saad et al., 2012; Wong et al., 2012), we used a regression of nuisance effects before bandpass filtering (RegBP; Hallquist et al., 2013). The data were despiked, and white matter (WM) and cerebrospinal fluid (CSF) components were regressed out as nuisance variables according to the aCompCor method. We then applied a linear detrending term. The residual BOLD time series went through a bandpass filter between 0.008 and 0.09 Hz to reduce the effect of low frequency drifts and high-frequency noise. All described steps are part of the standard procedure in the “conn” toolbox (Behzadi et al., 2007; Whitfield-Gabrieli and Nieto-Castanon, 2012). The residual head motion parameters (three rotation and three translation parameters, plus another six parameters representing their first-order temporal derivatives) were regressed out.

One pitfall of the analysis of resting state functional connectivity using seeds is the selection of seeds. The seed placement bias could lead to different and overlapping networks depending on the amount and placement of seeds (Cole et al., 2010). This bias can be reduced through the use of parcellations instead of spheres based on coordinates from the literature. We used a functional connectivity parcellation atlas based on a selection of parcels out of 330 parcels containing highly homogenous signal patterns (Gordon et al., 2014). This parcellation allowed us to perform two different analyses.

We first assessed functional connectivity on a seed based level. Two parcels were taken from the atlas of Gordon et al. (2014), localized at the structurally defined Heschl’s gyrus (left and right). These two seeds were chosen for their importance in auditory processing. With these seeds group analysis was performed to assess functional connectivity within both conditions as well as differences between the preferred music and control condition. Furthermore, first level beta maps were extracted (i.e., fisher transformed correlation values) for each participant and used to create individual figures for our *a priori* regions during both conditions (supplementary material). Data of healthy subjects were not directly compared to patients due to age differences, thus the difference between the music and control condition within one patient could not be compared to the range of differences within controls. Therefore, no within-subject statistical analysis was performed.

Although studies in healthy subjects show that single seeds can reveal whole networks, this is not necessarily the case in brain-damaged patients. Network disruption can be expected due to underlying neuropathology excluding regions from overall networks. To assess overall network characterization it is advised to use multiple seeds/regions (Demertzi et al., 2015). All parcels belonging to the auditory network, external network, and default mode network according to Gordon et al. (2014) were assessed for our group of patients in each condition. For all networks, time courses of the parcels were averaged and correlated to the whole brain (Halko et al., 2014; Demertzi et al., 2015). Thus, this averaged time series was used to estimate whole-brain correlation r maps, which were then converted to normally distributed Fisher’s z transformed correlation maps to allow for subsequent group-level analysis on the mean network connectivity (comparing music vs. control conditions). For all analyses on the group level (seed based and network based functional connectivity analysis) one sample *t*-tests were used for estimation of functional connectivity in each condition, and two-sample paired *t*-tests were used for between condition comparisons. The results were reported as significant when they exceeded a height threshold of uncorrected *p* = 0.001 with a family wise error corrected extent threshold of *p* = 0.05 at the cluster level. For clusters that showed significant stronger functional connectivity during the music condition contrast estimates (beta values) were extracted (Supplementary Figure S2). We did not compare the healthy group to our patient group due to differences in age, and the possible effects this might have on network integrity, as well as the possible differences in reaction to preferred music in terms of memory or emotion.

## Results

**FIGURE 1:**
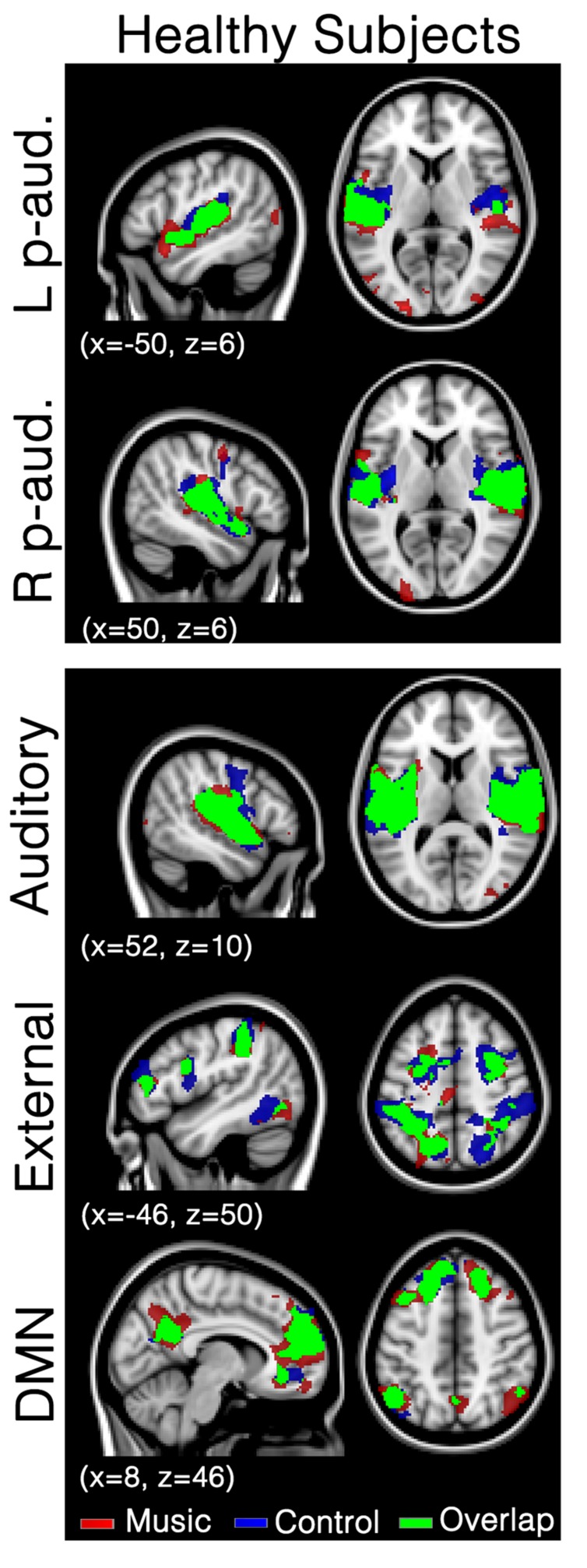
**Functional connectivity in healthy subjects during the music condition and the control condition.** Maps indicate healthy subjects’ (*N* = 8) functional connectivity during favorite music exposure (Red) and the control condition (Blue), and regions where functional connectivity was present in both conditions (Green). The top two panels show seed-based analyses, the lower three panels show mean network connectivity. Note that there is no significant difference between music and control condition. Results were analyzed in a network-based manner and thresholded with a family wise error corrected extended cluster level of *p* < 0.05. Standardized MNI T1 2x2x2 template was used to render results. (x,y,z) value indicates MNI coordinates of represented sections.

In healthy participants, seed-based analyses of both left and right primary auditory areas showed functional connectivity in areas considered as being part of the auditory network during both music and control conditions. Indeed, functional connectivity with seeds in both primary auditory cortices was observed in bilateral temporal gyri (encompassing Heschl’s gyrus, opercular gyrus, insula, planum polare, and superior temporal areas), anterior cingulate, pre- and post-central areas and the occipital pole (**Figure 1**; Supplementary Table S1) in both conditions. No significant difference was observed between the two conditions. Similarly, the auditory network showed activation in bilateral temporal gyri (encompassing Heschl’s gyrus, opercular, insula, planum polare, and superior temporal areas). This temporal cluster extended from inferior frontal, to precentral and angular areas. The auditory network also included the anterior cingulate, pre- and post-central areas and the occipital fusiform gyrus and cortex (**Figure 1**; Supplementary Table S2). The external network encompassed regions of bilateral inferior parietal sulcus and lobule, dorsolateral prefrontal, supramarginal, frontal eye field, lateral occipital and precentral, as well as cerebellar and insular areas. The default mode network showed functional connectivity with the precuneus, frontal pole and superior frontal gyrus, angular and lateral occipital gyrus, and middle temporal gyrus. For these three networks, the music condition did not significantly differ from the control condition.

**FIGURE 2:**
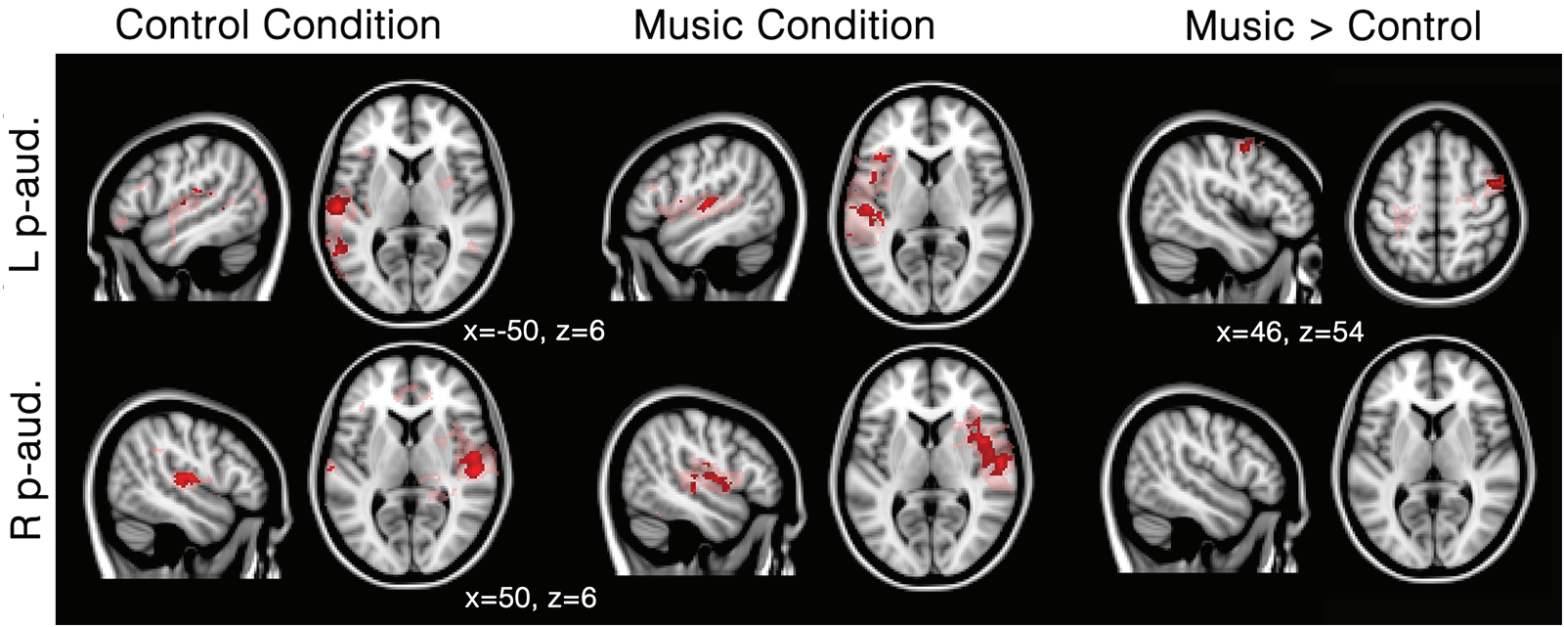
**Functional connectivity in patients during the music condition and the control condition using primary auditory seeds.** Red/pink maps indicate patients’ (*N* = 5) functional connectivity during the control condition (left) and favorite music exposure (middle) for both the left and right primary auditory cortex (L p-aud., and R p-aud.; respectively). Right maps show the regions that show significantly more functional connectivity during music condition compared to the control condition. Results were analyzed in a network-based manner and thresholded with a family wise error corrected extended cluster level of *p* < 0.05 (in red). For visualization a lowered threshold is indicated in pink (0.01 uncorrected height with family wise error corrected extended cluster level of *p* < 0.05). Standardized MNI T1 2x2x2 template was used to render results. (x,y,z) value indicates MNI coordinates of represented sections.

In patients, seed-based analyses of patients showed that functional connectivity was mainly restricted to the areas surrounding each of the two seeds (i.e., left and right primary auditory cortex) for both the music and the control conditions; however, several other clusters of functional connectivity were also observed (**Figure 2**; **Table 2**). The left primary auditory seed showed functional connectivity with the middle temporal gyrus during the control condition, and the left frontal operculum, superior temporal gyrus and cerebellum during the music condition. The right primary auditory seed showed several smaller clusters in the temporal area as well as the supramarginal area during the control condition, and one large cluster of activation in the temporal cortex during the music condition. When the music condition was directly compared to the control condition, the left primary auditory seed showed more functional connectivity in the right precentral gyrus during music. No difference was observed with the right primary auditory seed for this direct comparison. Single subject first level beta values (i.e., Fisher’s z transformed correlation values) were used to create individual patient figures for the two primary auditory seed activations during both conditions (Supplementary Figure S1). Correlation values during music and control conditions were mainly restricted to the areas surrounding each of the seeds, but in general, more voxels seemed to be strongly correlated in the music condition than in to the control condition (correlations higher than 0.8 were assessed and shown in the Supplementary Material).

**Table 2:** Results of the seed-based analyses in the patients.

	MNI coordinates (x,y,z)			Cluster size	Cluster p-FWE	p-unc peak	Region	
**Left primary auditory cortex**						
Music	-40	24	6	223	0	0.000003	Left	Frontal operculum
	-40	-26	6	200	0	0.000023	Left	Heschl/planum temporale
	-68	-36	14	52	0.025011	0.000007	Left	Superior temporal gyrus
	0	-48	-8	50	0.030795	0.000067		Cerebellum
Control	-60	-20	6	403	0	0	Left	Heschl/planum temporale
	-68	-46	4	163	0	0.000003	Left	Middle temporal gyrus



Music > Control	46	0	54	113	0.000007	0.000002	Right	Precentral gyrus
**Right primary auditory cortex**						
Music	40	26	10	886	0	0.000003	Right	Temporal cortex: insula/central opercular/planum temporale/Heschl/frontal operculum
Control	44	-16	10	379	0	0.000001	Right	Heschl gyrus/central opercular
	-68	-10	-2	85	0.00046	0.000047	Left	Superior temporal gyrus
	-64	-18	6	50	0.017807	0.000005	Left	Planum temporale
	28	-32	32	47	0.02515	0.000053	Right	Supramarginal gyrus

**FIGURE 3:**
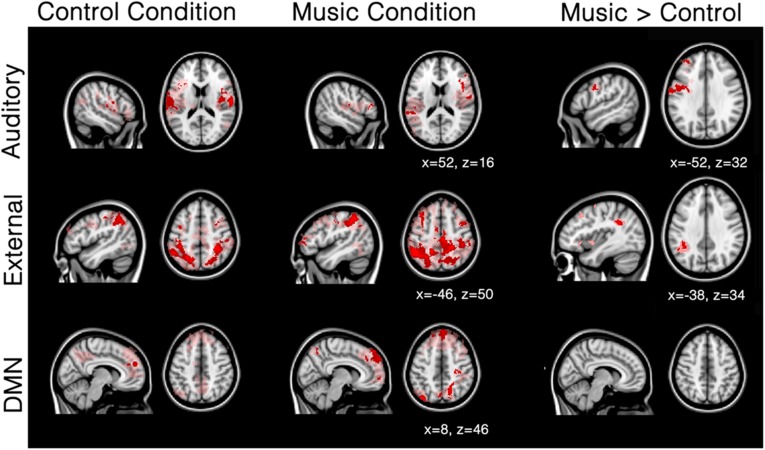
**Mean network connectivity in patients during the music condition and the control condition.** Red/pink maps indicate patients’ (*N* = 5) functional connectivity during the control condition (left) and favorite music exposure (middle) for the auditory network, external network, and default mode network (DMN). Right maps show the regions that show significantly more functional connectivity during music condition compared to the control condition. Results were analyzed in a network-based manner and thresholded with a family wise error corrected extended cluster level of *p* < 0.05 (in red). For visualization a lowered threshold is indicated in pink (0.01 uncorrected height with family wise error corrected extended cluster level of *p* < 0.05). Standardized MNI T1 2x2x2 template was used to render results. (x,y,z) value indicates MNI coordinates of represented sections.

Patients showed a severely limited auditory network of functional connectivity during both conditions (**Figure 3**; **Table 3A**). During the control condition, activation was only seen in bilateral temporal areas. During the music condition, the auditory network consisted of bilateral temporal gyri (only including left Heschl gyrus), as well as small clusters in the right inferior frontal gyrus and the left supramarginal gyrus; these were areas also included in the temporal cluster for the healthy subjects. When the music condition was compared to the control condition, the auditory network showed significantly more functional connectivity with the left precentral gyrus and a region on the junction of the middle frontal gyrus and frontal pole: the left dorsolateral prefrontal cortex.

**Table 3A:** Results of network-based analysis in patients: auditory network.

	MNI coordinates (x,y,z)			Cluster size	Cluster p-FWE	p-unc peak	Region	
**Auditory network**						
Music	-66	-40	14	161	0.000004	0.000034	Left	Supramarginal gyrus
	40	20	18	109	0.000174	0.000002	Right	Inferior frontal gyrus
	60	-4	12	97	0.000466	0.000032	Right	Temporal, central opercular
	-50	-30	20	48	0.042949	0.000507	Left	Parietal operculum/Heschl
Control	-50	-40	10	1152	0	0.000004	Left	Temporal cortex: planum temporale/central opercular/superior temporal
	28	6	2	997	0	0	Right	Temporal, central opercular/insula
	-36	20	12	208	0	0.000011	Left	Frontal operculum
	28	-26	26	46	0.046101	0.000094	Right	Parietal operculum



Music > Control	-66	-8	36	319	0	0.000001	Left	Precentral gyrus



	-28	42	30	44	0.028322	0.000019	Left	DLPFC

The external network in patients was restricted to the inferior parietal sulcus and lobule, dorsolateral, middle frontal, and supra marginal areas during both control and music conditions. (**Figure 3**; **Table 3B**). Compared to the control condition, music showed more functional connectivity with the supramarginal/angular gyrus, also referred to as the temporoparietal junction.

**Table 3B:** Results of network-based analysis in patients: external network.

	MNI coordinates (x,y,z)			Cluster size	Cluster p-FWE	p-unc peak	Region	
**External network**						
Music	58	-32	44	4974	0	0	Bilateral	Inferior parietal sulcus/inferior parietal lobule
	-36	24	52	424	0	0.000005	Left	DLPFC
	-52	32	16	122	0.000111	0.000008	Left	Middle frontal gyrus (small part FEF)
	-14	-10	64	116	0.000174	0.000013	Left	SMA
	48	12	56	100	0.000594	0.000021	Right	Middle frontal gyrus (small part FEF)
	30	34	-8	69	0.007915	0.000044	Right	DLPFC
	-38	-54	-12	59	0.019658	0.000013	Left	Lateral occipital/MT
	-56	-58	4	50	0.046263	0.000163	Left	Lateral occipital/MT
Control	-24	-62	48	2072	0	0.000001	Left	Inferior parietal sulcus/inferior parietal lobule
	12	-74	54	1026	0	0.000004	Right	Inferior parietal sulcus/inferior parietal lobule
	-32	14	24	403	0	0.000003	Left	SMA extending to small part FEF
	-42	48	24	104	0.000223	0.000012	Left	DLPFC
	34	8	52	82	0.001473	0.000065	Right	Middle frontal gyrus (small part FEF)



Music > Control	-42	-50	30	103	0.000078	0.000003	Left	Supramarginal/angular gyrus

The default-mode network in patients seemed disconnected in patients (**Figure 3**; **Table 3C**). The control condition only showed functional connectivity in the frontal pole/paracingulate gyrus. The music condition showed further functional connectivity with the precuneus, post-central gyrus, lateral occipital pole, and middle temporal gyrus. However, no difference could be found between the two conditions.

**Table 3C:** Results of network-based analysis in patients: default mode network.

	MNI coordinates (x,y,z)			Cluster size	Cluster p-FWE	p-unc peak	Region	
**Default mode network**						
Music	-26	32	34	1247	0	0.000001	Bilateral	Middle frontal gyrus/frontal pole/paracingulate gyrus
	12	-66	62	233	0	0.000004	Right	Precuneus/lateral occipital
	-38	-76	48	150	0.000014	0.000001	Left	Lateral occipital
	-30	52	2	110	0.000264	0.000124	Left	Frontal pole
	-58	-24	-12	81	0.002724	0.000004	Left	Middle temporal gyrus
	8	60	-4	56	0.025536	0.000149	Right	Frontal pole
	28	-24	46	53	0.034	0.000094	Right	Post-central gyrus
Control	-10	48	18	679	0	0.000034	Left	Frontal pole/paracingulate gyrus

## Discussion

In the present study, we aimed at assessing the potential effect of music on the brain’s functional connectivity in patients with DOC. We compared patients’ intrinsic brain activation while being exposed to their preferred music and during a control condition. For this purpose, seed-based functional connectivity as well as network-level functional connectivity was assessed. Seed-based functional connectivity analyses of primary auditory cortices showed significant differences in functional connectivity between music and control conditions for the patients. Network-level analyses showed that patients’ functional connectivity is increased when being exposed to their preferred music in the auditory and external network (in comparison to the control condition).

In healthy participants, the network of functional connectivity based on both primary auditory regions encompasses large parts of the auditory cortex, superior temporal gyri, insula, cingulate cortex, central areas (pre and post), supramarginal gyrus, and occipital areas (**Figure 1**), in both the music condition and the control condition. These are, as expected, part of the auditory network (Beckmann et al., 2005; Damoiseaux et al., 2006; De Luca et al., 2006; Smith and Tindell, 2009; Laird et al., 2011; Demertzi et al., 2014). To assess network integrity, mean network connectivity was assessed in the auditory network, external network, and default mode network, i.e., networks that are respectively linked to auditory processing, external orientation, and internal thoughts.

Network-based second level analysis of functional connectivity showed that the auditory network was clearly replicated in our healthy subjects during both the music and control conditions. This network has consistently been observed in previous resting state studies investigating not only healthy participants but also DOC patients (Demertzi et al., 2014). In healthy participants it encompassed bilateral temporal gyri (including Heschl’s gyrus, opercular, insula, planum polare, and superior temporal areas), extending to inferior frontal, precentral and angular areas, as well as clusters in anterior cingulate, pre- and post-central areas and the occipital fusiform gyrus (Beckmann et al., 2005; Damoiseaux et al., 2006; De Luca et al., 2006; Smith and Tindell, 2009; Laird et al., 2011; Demertzi et al., 2014). The external network has also been observed in healthy participants. It encompassed, as consistently observed in previous studies (Fox et al., 2005; Vanhaudenhuyse et al., 2010a), regions of bilateral inferior parietal sulcus and lobule, dorsolateral prefrontal, supramarginal gyrus, the frontal eye field, lateral occipital and precentral, as well as cerebellar and insular areas. The default-mode network showed functional connectivity in regions consistently observed in healthy participants and patient populations (Buckner et al., 2008). Most importantly, music did not show any increases in functional connectivity compared to the control condition for the seed-based and all three network-level analyses. This result is consistent with Castro et al. (2015) who observed that music (in comparison to noise) did not modify the event-related responses in healthy participants (while this was the case for the DOC patients). This observation suggests that the effects of music observed in previous research are possibly not present in healthy subjects (or that the cerebral responses could not be enhanced because they were already at ceiling). This finding could be due to the nature of our experimental material. Indeed Wilkins et al. (2014) have shown functional connectivity differences (in the default mode network and between auditory brain areas and the hippocampus) between two music materials that strongly differ in terms of emotion, i.e., preferred and disliked music (in healthy participants). It is thus possible, that our control condition, which can be considered as rather neutral, was not disliked enough to warrant significant differences in functional connectivity with the preferred music condition.

Seed-based analysis indicated that patients showed strongly limited functional correlations with the primary auditory cortices: activation was only observed around the seed areas and no long distance connectivity emerged within the auditory network. This finding is in line with previous research showing a linear decrease in functional connectivity ranging from healthy participants to unresponsive patients (Vanhaudenhuyse et al., 2010b; Thibaut et al., 2012; Demertzi et al., 2014). In fact, many studies have shown that functional connectivity still exists in DOC patients, and other forms of decreased levels of consciousness (Heine et al., 2012). Low-level activations in primary auditory cortices, without top–down feedback have also been observed in unresponsive patients (Laureys et al., 2000; Boly et al., 2011). In fact, patients seem to have a general disconnection between brain regions, notably missing long range connectivity (Casali et al., 2013). Our results are congruent with this observation as we observe mainly functional connectivity in the hemisphere of the seed. Furthermore, significant differences in the right precentral gyrus are observed during the preferred music condition compared to the control condition (**Figure 2**). This finding is in agreement with a previous study investigating DOC patients and reporting activation in the right superior temporal gyrus during three 10-s blocks of musical stimulation based on a famous song (Okumura et al., 2014).

First-level connectivity maps of each patient suggest larger areas of correlation near the seed during the music condition than during the control condition (Supplementary Figure S1). This difference seems to be present for all subjects, even the subjects clinically diagnosed as UWS (DOC1 and 2). This finding fits with the neuroimaging results observed in DOC1: diagnostic assessment based on PET metabolism suggested MCS (e.g., Stender et al., 2014). However, the second patient who was diagnosed as UWS (DOC2) both clinically and using neuroimaging, also showed more voxels correlated to the seed, indicating that the effect of music as reported here (if replicable in future studies with extended patient samples) might be present for all DOC. It is important to note that stronger correlating voxels were observed during the music condition (as compared to the control condition) in all patients for at least one seed. Also, no clear correlation with etiology, or time since injury can be seen due to the limited sample.

The three network analyses further revealed significant differences in the auditory network and external network, but not the default mode network, during the music condition. Patients showed a severely limited auditory network of functional connectivity during both conditions (**Figure 3**). During the control condition, activation was only seen in bilateral temporal areas. During the music condition, the auditory network was restricted to bilateral temporal gyri (only left including Heschl’s gyrus) and small clusters in the right inferior frontal gyrus and the left supramarginal gyrus, areas included in the temporal cluster for the healthy subjects. The right inferior frontal gyrus is implicated in auditory memory as well as the processing of musical syntactic-like structures (Maess et al., 2001; Janata et al., 2002; Koelsch et al., 2002, 2005; Tillmann et al., 2003, 2006; Koelsch and Siebel, 2005; Albouy et al., 2013). When music was compared to the control condition, patients’ auditory network showed significantly more functional connectivity with the left precentral gyrus (Note that the seed-based analysis also revealed significant increased functional enhancement in the right precentral gyrus during music; see **Figure 2**) and the left frontal pole. The precentral cluster overlaps with regions of the auditory network in healthy subjects. The lateral prefrontal cortex has also been linked to autobiographical memory (Svoboda et al., 2006; Cabeza and St Jacques, 2007), and has also been implicated in rhythm perception (Zatorre et al., 2007). The finding of increased functional connectivity in music compared to the control condition suggests that music has an effect on the auditory-related network in DOC patients, in whom short-term functional plasticity might appear following the lesions.

In patients, the external network observed during the control condition was restricted to clusters of functional connectivity in inferior parietal sulcus and lobule, dorsolateral, middle frontal, and supramarginal areas. In the music condition, the external network showed besides these regions also connectivity with the region MT and parts of the frontal eye field. When directly compared to the control condition, the music condition showed more functional connectivity with the supramarginal/angular gyrus. This cluster overlaps with the supramarginal regions activated during spatial orienting in healthy subjects (Corbetta and Shulman, 2002). Interestingly, this region overlaps with disconnected areas in UWS patients (Laureys et al., 2000). Laureys et al. (2000) proposed that a lack of integration between primary regions (that activate after simple auditory stimulations in UWS), and higher order regions like the temporoparietal junction and superior temporal gyri (activated in MCS after simple auditory stimuli; Boly and Faymonville, 2004) makes conscious processing unlikely (Laureys et al., 2000; Boly and Faymonville, 2004). Put differently, unconsciousness might be related to a disruption in feedback processing to the auditory regions (Boly et al., 2011).

## Conclusion

The effect of music on functional cerebral connectivity is reminiscent of previous findings which have shown effects of music in brain-damaged patients (Soto and Funes, 2009; Särkämö and Soto, 2012; Verger et al., 2014; Castro et al., 2015). For example, a recent EEG study investigating DOC patients has shown that the patients’ cerebral responses following the presentation of one’s own name were increased after having been exposed to their preferred music (Castro et al., 2015). A “Mood and Arousal hypothesis,” attributes the beneficial effects of music on cognition to an increase in mood and arousal (Chabris, 1999; Nantais and Schellenberg, 1999). Within this hypothesis, the effects of music in DOC patients might be due to an overall cortical arousal in the cerebral structures that have been reported to be involved in emotional and mood states. A second hypothesis attributes the effect of music to autobiographical priming (Castro et al., 2015). Interestingly, in the present study, an increased functional connectivity during the music condition (vs. the control condition) was shown in cortical structures linked to music perception, autobiographical memory and consciousness for DOC patients. These results need to be confirmed in an extended group of patients, and future studies should also disentangle the general effect of music (because of its acoustic and structural features) from its autobiographical effects (because of its emotional and meaningful contents in relation to the patients’ personal memory).

## Conflict of Interest Statement

The authors declare that the research was conducted in the absence of any commercial or financial relationships that could be construed as a potential conflict of interest.

## Footnotes

1 www.fil.ion.ucl.ac.uk/spm

2 http://www.nitrc.org/projects/artifact\_detect/

3 http://www.nitrc.org/projects/conn
