# Supplementary material for: Exploration of Functional Connectivity During Preferred Music Stimulation in Patients with Disorders of Consciousness
Source: Front Psychol. 2015 Nov 9;6:1704. doi: 10.3389/fpsyg.2015.01704 (PMC4637404; doi:10.3389/fpsyg.2015.01704)
Supplement: Supplementary file 1 [file Data_Sheet_1.DOCX]

**Supplementary Material**

Figure 1: Single subject connectivity Single subject first level beta value maps (e.g. fisher transformed correlation values) of both conditions were rendered on each subject’s own T1 brain image. Beta values from .8 up are shown. Red maps indicate the music condition; blue maps indicate the control condition; green maps indicate functional connectivity during both the music and noise condition. Note that more voxels seem to be more strongly correlated to areas surrounding the seed (temporal gyri) during the music condition than to the control condition.


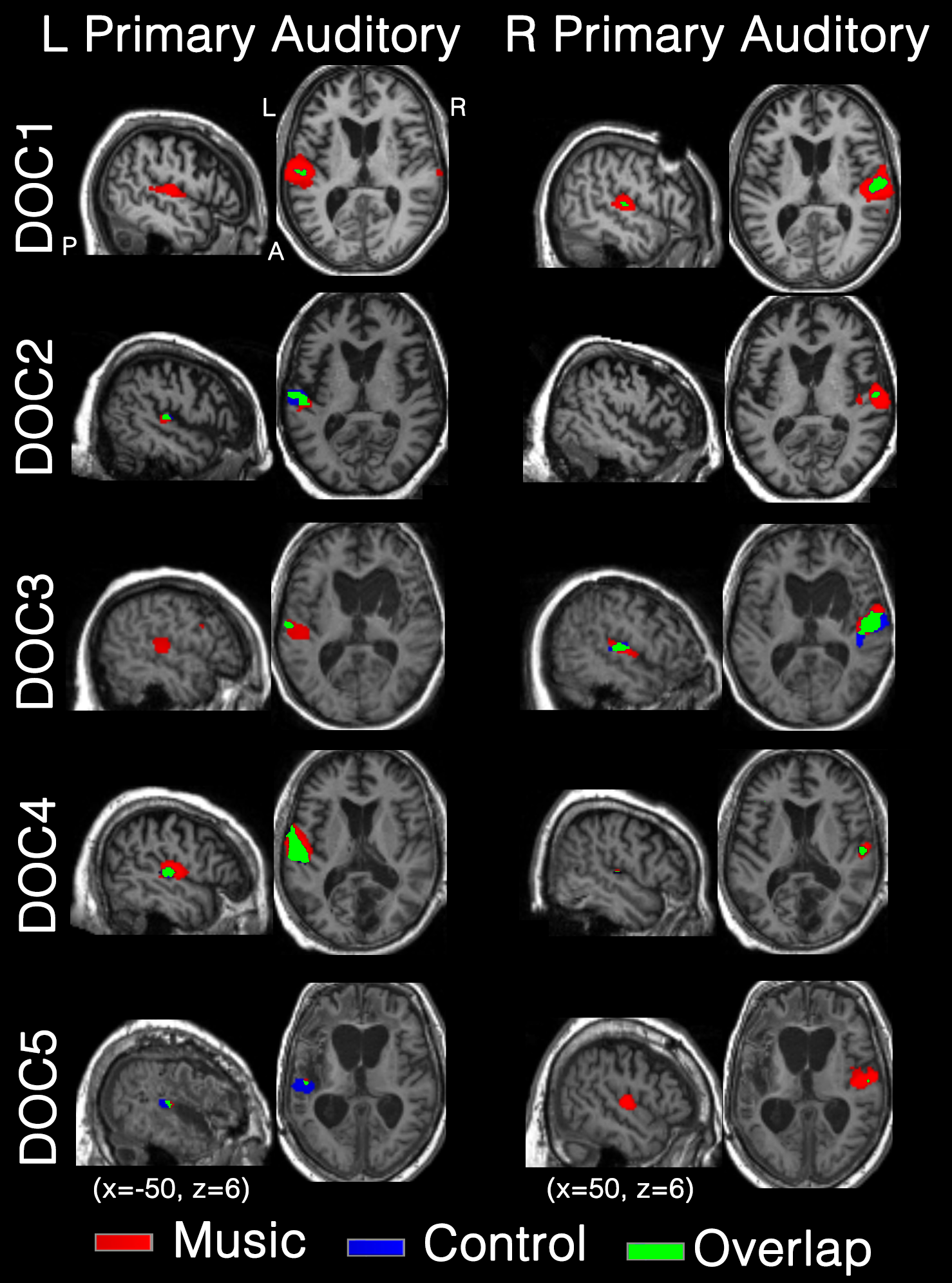


Figure 2: Contrast estimates. We have extracted the contrast estimates (beta values) for our significant clusters (music>control condition) in all our analysis. Beta values of the precentral area were not significantly different from zero during the control condition, while they were significantly positive during the music condition. This was also the case for both the left primary auditory seed (music beta=0.17; pFDR=0.005, control beta=-0.04; pFDR=0.321), and the auditory network (music beta=0.12; pFDR=0.0349, control beta=-0.03; pFDR=0.528). The auditory network also shows significant differences (music>control condition) with the dorsolateral prefrontal cortex, and the same effect was observed here (music beta=0.05; pFDR=0.0349, control beta=-0.04; pFDR=0.139). The external network showed significant differences in the supramarginal/angular gyrus. Functional connectivity was significantly positive in this cluster in both the control and music condition, but stronger during the music condition (music beta=0.12; pFDR=0.0005, control beta=0.03; pFDR=0.0256).


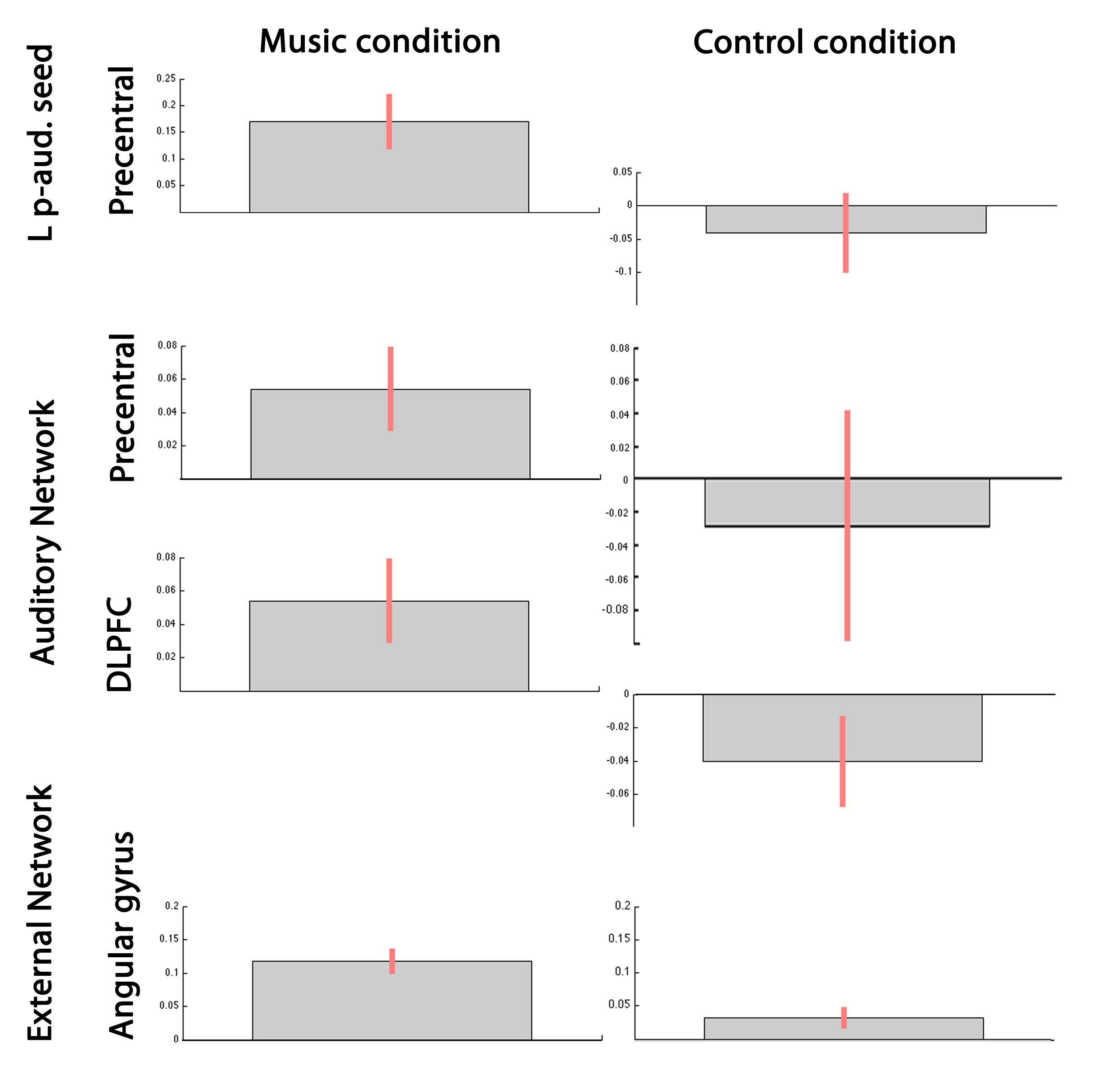


**Table 1 Results seed based analysis in healthy subjects**

|  | Left Primary auditory | | | | | | | |
| --- | --- | --- | --- | --- | --- | --- | --- | --- |
|  | **MNI coordinates**  **(x,y,z)** | | | **Cluster size** | **Cluster**  **p-FWE** | **p-unc**  **peak** | **Region** |  |
| Music | -44 | -28 | 12 | 3712 | 0 | 0 | Left | Left planum temporale / temporal pole / central opercular / Heschl |
|  | 54 | -4 | -10 | 2692 | 0 | 0 | Right | Temporal pole / planum polare / superior temporal gyrus / insula / Heschl |
|  | -6 | -10 | 24 | 1076 | 0 | 0 | Left | Occipital pole |
|  | 34 | -88 | 22 | 272 | 0.000014 | 0 | Right | Occipital pole / lateral occipital cortex |
|  | 38 | -56 | -20 | 238 | 0.00005 | 0.000006 | Right | temporal occipital / fusiform gyrus |
|  | -44 | -74 | 12 | 205 | 0.000183 | 0.000045 | Left | Lateral occipital cortex |
|  | -36 | 24 | -4 | 138 | 0.003221 | 0.000003 | Left | Frontal orbital cortex / insular cortex |
|  | -36 | -80 | -16 | 110 | 0.012177 | 0.000009 | Left | Occipital fusiform gyrus |
|  | 16 | -80 | -12 | 84 | 0.04594 | 0 | Right | Lingual gyrus |
| Control | -58 | -18 | 6 | 3624 | 0 | 0 | Left | Insula / planum temporale / central opercular / parietal operculum / heschl / planum polare |
|  | 54 | -6 | 0 | 1534 | 0 | 0.000001 | Right | Planum polare / insula / temporal pole / heschl / central opercular / superior temporal gyrus |
|  | 8 | 2 | 36 | 540 | 0 | 0.000001 | Left | Cingulate gyrus |
|  | 52 | 0 | 44 | 295 | 0.000082 | 0.000018 | Right | Precentral gyrus |
|  | 56 | -30 | 18 | 207 | 0.001239 | 0.000107 | Right | Planum temporale / parietal operculum |
|  | -18 | -28 | 42 | 162 | 0.00573 | 0.000033 | Left | Pre and postcentral gyrus |
|  | 16 | -40 | 62 | 150 | 0.008813 | 0.000001 | Right | Pre and postcentral gyrus |
|  | 12 | -20 | 42 | 116 | 0.031731 | 0.000007 | Right | Cingulate / precentral gyrus |

|  | Right Primary auditory | | | | | | | |
| --- | --- | --- | --- | --- | --- | --- | --- | --- |
|  | **MNI coordinates**  **(x,y,z)** | | | **Cluster size** | **Cluster**  **p-FWE** | **p-unc**  **peak** | **Region** | |
| Music | 52 | -18 | 10 | 4352 | 0 | 0 | Right | Central opercular / planum temporale / temporal pole / superior temporal gyrus / heschl / insula / frontal orbital / superior temporal gyrus |
|  | -58 | 10 | 10 | 3256 | 0 | 0.000001 | Left | Central opercular / parietal operculum / planum temporale / insula / heschl / planum polare / recentral gyrus / superior temporal gyrus |
|  | -28 | -58 | -20 | 1150 | 0 | 0.000002 | Left | occipital fusiform gyrus / temporal occipital / cerebelum / lingual gyrus |
|  | 2 | -10 | 28 | 587 | 0 | 0.000001 | Right | Cingulate gyrus / suplementary motor cortex |
|  | -14 | -18 | -12 | 447 | 0 | 0 | Left | Amygdala |
|  | -20 | -96 | 12 | 427 | 0 | 0.000021 | Left | Occipital pole |
|  | -44 | -12 | 48 | 269 | 0.000046 | 0.000036 | Left | precentral gyrus |
|  | -16 | -88 | 46 | 263 | 0.000056 | 0.000027 | Left | Lateral occipital |
|  | 48 | -2 | 44 | 182 | 0.001049 | 0.000001 | Right | Precentral gyrus |
|  | 0 | -8 | 66 | 114 | 0.017382 | 0.0001 | Right | Suplementary motor cortex. |
| Control | 60 | -28 | 14 | 5100 | 0 | 0 | Right | Central opercular / insula / planum temporale / parietal operculum, temporal pole / heschl / precentral gyrus |
|  | -42 | -16 | 6 | 3822 | 0 | 0.000001 | Left | Central opercular / insula / planum temporale / parietal operculum / heschl / planum polare |
|  | 16 | -38 | 54 | 298 | 0.000182 | 0.000016 | Right | post and precentral gyrus |

**Table 2 : Network based analysis in healthy subjects**

|  | Auditory Network | | | | | | | |
| --- | --- | --- | --- | --- | --- | --- | --- | --- |
|  | **MNI coordinates**  **(x,y,z)** | | | **Cluster size** | **Cluster**  **p-FWE** | **p-unc**  **peak** | **Region** |  |
| Music | -8 | -54 | 60 | 4021 | 0 | 0 | Left | inferior parial sulcus / inferior parietal lobule |
|  | 32 | -76 | 38 | 1443 | 0 | 0 | Right | inferior parial sulcus / inferior parietal lobule |
|  | 58 | 12 | 38 | 1328 | 0 | 0 | Right | DLPFC |
|  | -12 | -2 | 54 | 1083 | 0 | 0 | Left | SMA / Pre-SMA |
|  | -52 | -74 | -16 | 955 | 0 | 0 | Left | Lateral occipital / MT |
|  | -44 | 32 | 12 | 507 | 0.000001 | 0 | Left | DLPFC |
|  | 30 | -10 | 46 | 478 | 0.000001 | 0 | Right | FEF |
|  | 60 | -58 | -26 | 359 | 0.000027 | 0.000001 | Right | Lateral occipital / MT |
| Control | -22 | -42 | 52 | 5449 | 0 | 0 | Left | inferior parial sulcus / inferior parietal lobule |
|  | 38 | -30 | 62 | 5257 | 0 | 0 | Right | inferior parial sulcus / inferior parietal lobule |
|  | 30 | 10 | 40 | 3035 | 0 | 0 | Right | SMA / Pre-SMA |
|  | -12 | -10 | 66 | 1845 | 0 | 0 | Left | SMA / Pre-SMA |
|  | -36 | 36 | 32 | 825 | 0 | 0 | Left | DLPFC |

|  | Dorsal attention Network | | | | | | | |
| --- | --- | --- | --- | --- | --- | --- | --- | --- |
|  | **MNI coordinates**  **(x,y,z)** | | | **Cluster size** | **Cluster**  **p-FWE** | **p-unc**  **peak** | **Region** |  |
| Music | -8 | -54 | 60 | 4021 | 0 | 0 | Left | inferior parial sulcus / inferior parietal lobule |
|  | 32 | -76 | 38 | 1443 | 0 | 0 | Right | inferior parial sulcus / inferior parietal lobule |
|  | 58 | 12 | 38 | 1328 | 0 | 0 | Right | DLPFC |
|  | -12 | -2 | 54 | 1083 | 0 | 0 | Left | SMA / Pre-SMA |
|  | -52 | -74 | -16 | 955 | 0 | 0 | Left | Lateral occipital / MT |
|  | -44 | 32 | 12 | 507 | 0.000001 | 0 | Left | DLPFC |
|  | 30 | -10 | 46 | 478 | 0.000001 | 0 | Right | FEF |
|  | 60 | -58 | -26 | 359 | 0.000027 | 0.000001 | Right | Lateral occipital / MT |
|  | -56 | 6 | 34 | 271 | 0.000293 | 0.00001 | Left | FEF |
|  | 2 | -88 | -34 | 238 | 0.000763 | 0.000025 |  | cerebellum |
|  | -6 | -28 | 50 | 168 | 0.006817 | 0.000227 | Left | precentral / cingulate |
| Control | -22 | -42 | 52 | 5449 | 0 | 0 | Left | inferior parial sulcus / inferior parietal lobule |
|  | 38 | -30 | 62 | 5257 | 0 | 0 | Right | inferior parial sulcus / inferior parietal lobule |
|  | 30 | 10 | 40 | 3035 | 0 | 0 | Right | SMA / Pre-SMA |
|  | -12 | -10 | 66 | 1845 | 0 | 0 | Left | SMA / Pre-SMA |
|  | -36 | 36 | 32 | 825 | 0 | 0 | Left | DLPFC |
|  | 50 | 50 | 14 | 778 | 0 | 0 | Right | DLPFC |
|  | -44 | -48 | -16 | 775 | 0 | 0 | Left | Lateral occipital / MT |
|  | -38 | 14 | 14 | 657 | 0 | 0 | Left | FEF |
|  | 48 | -50 | -16 | 482 | 0.000001 | 0 | Right | Lateral occipital / MT |
|  | -36 | -12 | 0 | 117 | 0.040694 | 0.001383 | Left | insula |

|  | Default mode network | | | | | | | | |
| --- | --- | --- | --- | --- | --- | --- | --- | --- | --- |
|  | **MNI coordinates**  **(x,y,z)** | | | **Cluster size** | **Cluster**  **p-FWE** | **p-unc**  **peak** | **Region** |  | |
| Music | 18 | 26 | 32 | 11044 | 0 | 0.000007 | Left | | Frontal pole / superior frontal gyrus / paracingulate gyrus / cingulate gyrus / |
|  | 4 | -64 | 24 | 2420 | 0 | 0.000042 | bilateral | | Precuneus |
|  | -54 | -56 | 52 | 2025 | 0 | 0.000024 | Left | | Lateral occipital gyrus / angular gyrus |
|  | 56 | -60 | 28 | 1468 | 0 | 0.000062 | Right | | Angular gyrus / lateral occipital gyrus |
|  | 24 | 12 | -36 | 966 | 0 | 0.000074 | Right | | Temporal pole / middle temporal gyrus / frontal orbital cortex |
|  | -56 | -28 | -4 | 427 | 0.000013 | 0.000208 | Left | | Middle temporal gyrus |
|  | -30 | 16 | -18 | 327 | 0.000142 | 0.000089 | Left | | Temporal pole |
|  | -44 | 32 | -20 | 292 | 0.000343 | 0.000127 | Left | | Frontal orbital cortex |
|  | 16 | -92 | -30 | 166 | 0.011743 | 0.000257 |  | | Cerebellum |
| Control | -22 | -42 | 52 | 9211 | 0 | 0.000005 | Left | | Frontal pole / superior frontal gyrus / frontal pole / paracingulate gyrus/ middle frontal gyrus / paracingulate gyrus / cingulate gyrus |
|  | 38 | -30 | 62 | 1781 | 0 | 0.000027 | Left | | Precuneus / cingulate gyrus |
|  | 30 | 10 | 40 | 1609 | 0 | 0.000072 | Left | | Lateral occipital / angular gyrus |
|  | -12 | -10 | 66 | 1236 | 0 | 0.000009 | Left | | Temporal pole / middle temporal gyrus |
|  | -36 | 36 | 32 | 1118 | 0 | 0.000009 | Right | | Temporal pole / middle temporal gyrus |
|  | 50 | 50 | 14 | 664 | 0 | 0.000202 | Right | | angular gyrus / lateral occipital cortex |
|  | -44 | -48 | -16 | 123 | 0.041643 | 0.000237 | Bilateral | | cingulate gyrus |
