# Supplementary figures and images for: Exploration of Functional Connectivity During Preferred Music Stimulation in Patients with Disorders of Consciousness
Source: Front Psychol. 2015 Nov 9;6:1704. doi: 10.3389/fpsyg.2015.01704 (PMC4637404; doi:10.3389/fpsyg.2015.01704)

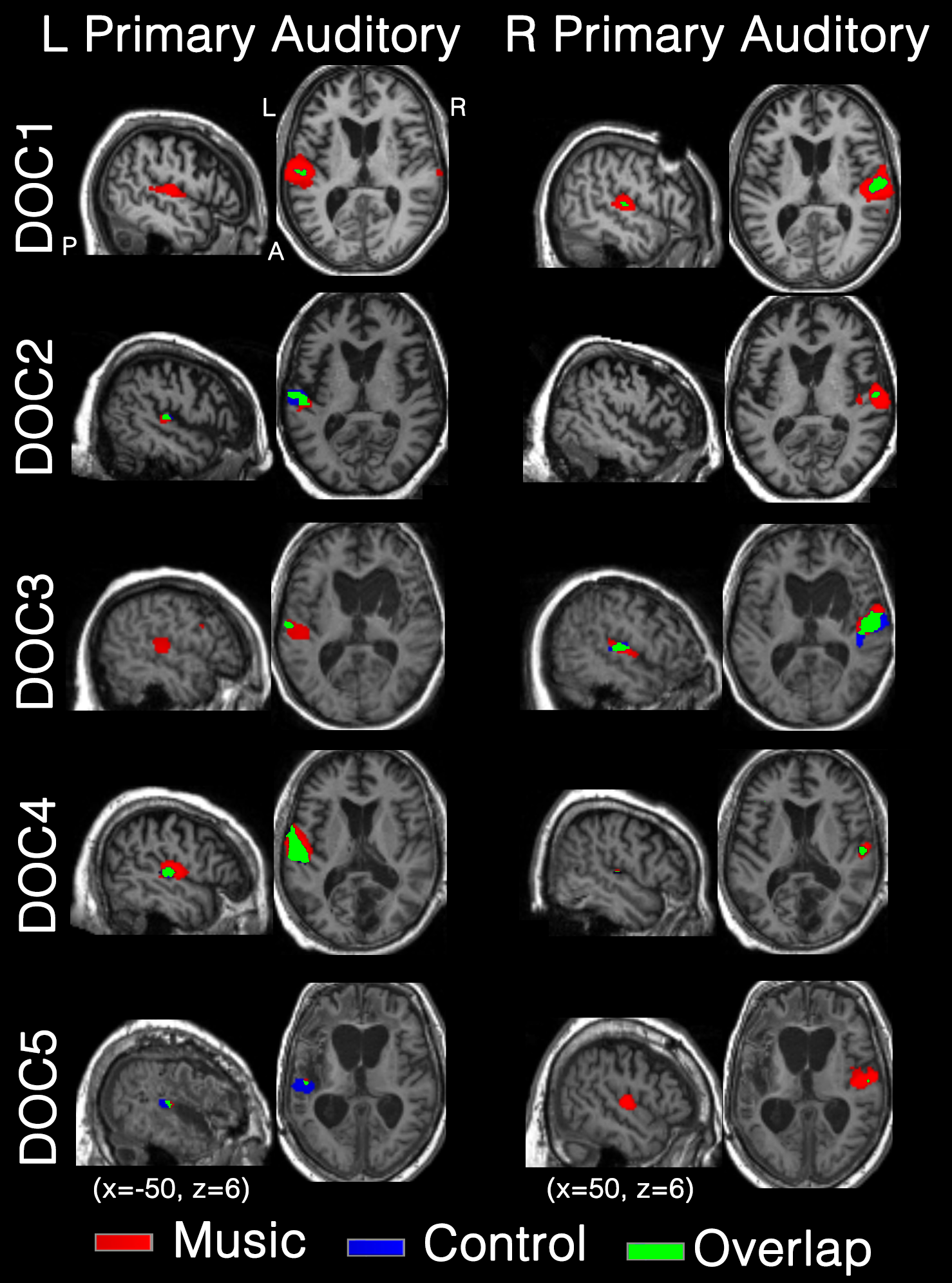

Supplement: Supplementary file 2 [file Image_1.TIF]

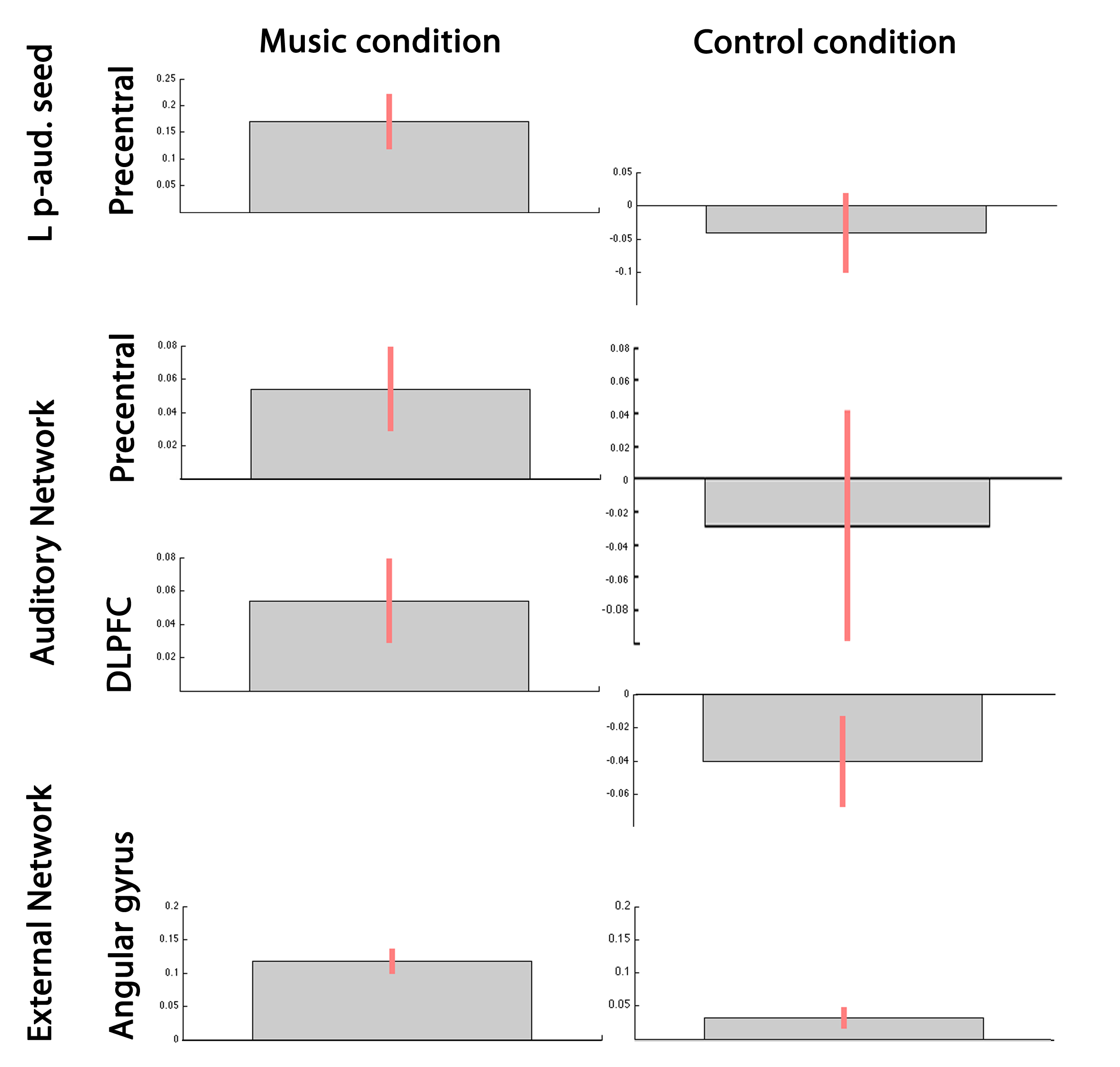

Supplement: Supplementary file 3 [file Image_2.TIF]
